# Association of *DEAR1* Tagging Single Nucleotide Polymorphisms With Breast Cancer in a Sample of Colombian Population: A Case Control Study

**DOI:** 10.1177/1178223420904939

**Published:** 2020-04-20

**Authors:** Angela P Beltrán, Edgar Benitez, Martin Rondon, Yeimy V Ariza, Fabio A Aristizabal, Ignacio Briceño

**Affiliations:** 1 Biotechnology Institute, Universidad Nacional de Colombia, Bogotá, Colombia; 2 Institute of Genetic, Faculty of Medicine, Pontificia Universidad Javeriana, Bogotá, Colombia; 3 Faculty of Medicine, Universidad de la Sabana, Chía, Colombia; 4 Department of Clinical Epidemiology and Biostatistics, Pontificia Universidad Javeriana, Bogotà, Colombia; 5 Pharmacology Department, Universidad Nacional de Colombia, Bogotá, Colombia

**Keywords:** Breast cancer, *DEAR1*, genetic susceptibility, polymorphism, ubiquitins, HER, progesterone receptor

## Abstract

**Purpose:**

Ubiquitin ligase genes can act as oncogenes or tumor suppressor genes. They play a role in various diseases, including development and progression of breast cancer; the objective of this study was to evaluate the association of common variants in the *ductal-epithelium-associated RING chromosome 1* (*DEAR1*) gene with breast cancer risk in a sample of Colombian population.

**Methods:**

We carried out a case-control study to investigate associations of variants in *DEAR1* with breast cancer in women from Colombia. Single nucleotide polymorphisms (SNPs) rs584298, rs2927970, rs59983645, and rs599167 were genotyped in 1022 breast cancer cases and 1023 healthy controls using the iPLEX® and Kompetitive Allele Specific PCR (polymerase chain reaction) (KASP) method. The associations between SNPs and breast cancer were examined by conditional logistic regression. The associations between SNPs and epidemiological/histopathological variables were examined by multinomial logistic regression.

**Results:**

Associations were found between tag SNPs and breast cancer adjusted for the epidemiological risk factors rs584298 genotypes AG and GG (*P* = .048 and *P* = .004, respectively). The analysis of the disease characteristics showed that SNP rs584298 (genotype AG) (*P* = .015) shows association with progesterone receptor (PR) status and (genotype AA) (*P* = .048) shows association with human epidermal growth factor receptor 2 (HER2) status.

**Conclusions:**

The SNP rs584298 in *DEAR1* showed associations with breast cancer and the expression of HER2 receptor; when this receptor is amplified, the result is aggressive tumoral subtype and expression of PR receptor that is associated with high-proliferative tumor grade. Validation of this SNP is important to establish whether this variant or the tagged variant is the cause for the risk association showed.

## Introduction

Breast cancer is the most common cancer in women worldwide and is the leading cause of death among cancer cases. Different genetic and nongenetic factors are associated with risk of breast cancer. Ubiquitin ligase proteins are engaged in the regulation of the turnover and the activity of many target proteins. The *ductal-epithelium-associated RING chromosome 1* gene (*DEAR1*) has 5 exons; it is located in chromosome 1p35,^1^ a region with a high frequency of loss of heterozygosity in different types of tumors.^2^ It encodes an E3 ubiquitin-protein ligase. The members of this family participate in several cellular processes, such as proliferation, differentiation, oncogenesis, apoptosis, intracellular trafficking, innate cellular responses to retroviral infection, and inflammation. The *DEAR1* gene in breast cancer has been considered a predictor of local recurrence-free survival in early-onset breast cancer; it regulates polarity and tissue architecture^1^ and is a master regulator of transforming growth factor β (TGF-β)-driven epithelial-mesenchymal transition (EMT).^3^ In breast cancer, *DEAR1* is downregulated in ductal carcinoma in situ lesions and infiltrating ductal carcinoma.^1^

In total, 230 single nucleotide polymorphisms (SNPs) have been identified based on genotype data from 60 CLM individuals (CLM: Medellin-Colombia) in the 1000 Genomes Project,^4^ different to the genotype data from 116 CEU individuals (CEU: Utah residents with ancestry from northern and western Europe) that contain 94 SNPs in HapMap (haplotype map).^5^ This difference confirms that SNP frequencies change across different populations, and it is important to define which SNPs are associated with risk in each population. Most of the breast cancer cases are sporadic, and in the 10% of the hereditary cancer, *BRCA1* and *BRCA2* (breast cancer genes 1 and 2) are the best-known susceptibility genes; they are associated with the risk of up to 85% of developing breast cancer in mutation carriers.^6^,^7^

Although the impact of high-risk mutations is important, they account for less than 25% of the cases of breast cancer predisposition; other risk factors can be explained by the addition of multiple common variants, known as low-penetrance variants in the polygenic model.^8-11^ Analyses performed on different populations allow discovering causal factors.^12^ The association of Tag SNPs rs584298, rs2927970, rs59983645, and rs599167 in *DEAR1* with risk of breast cancer was analyzed in the total of cases and controls in women from the Colombian population by genotyping. This is the first study conducted on the association between SNPs in *DEAR1* and breast cancer.

## Methods

### Study design

Case-control study was carried out; cases were patients with breast cancer with different histopathological reports, and controls were patients without breast cancer or any breast injury.

### Patients and controls

#### Patients

In total, 1022 cases were included. Patients with breast cancer were enrolled at different hospitals throughout the country, mainly at hospitals in Bogotá, Neiva, and Villavicencio. Unselected patients (all the patients who assisted to oncology appointment with breast cancer diagnosis) were recruited during the period 03/2007 to 02/2011 within the Col-BCCC (Colombian breast cancer case-control study), at PUJ (Pontificia Universidad Javeriana Bogotá-Colombia)^13^ Cases were women with a diagnosis of breast cancer after January 1, 2004. Controls were recruited in the period of 06/2007 to 06/2011. Clinical, epidemiological, and histopathological data were collected as well as informed consent. The data included age at breast cancer diagnosis, histology, histological grade, tumor size, lymph node status, estrogen receptor (ER) status, progesterone receptor (PR) status, and human epidermal growth factor receptor 2 (HER2) status; epidemiological data included menopausal status, family history of breast and ovarian cancer, postmenopausal hormone therapy, oral contraceptive (OC) use, body mass index (BMI), and smoking. Data on follow-up of the breast cancer patients were collected ±5 years after the date of breast cancer diagnosis during the course of this study, including the date of last follow-up, date of locoregional relapse, date of distant metastasis relapse, date of contralateral breast cancer, date of death, and cause of death. This study was approved with the code MED-119-2009 by the ethics committee of Pontificia Universidad Javeriana.

#### Controls

In total, 1022 controls were included. The participants were healthy and unrelated women, who participated in the National Pap Smear Program.^14^ All study participants gave written informed consent. Controls were matched to cases with a range of up to 2 years.

### Eligibility criteria

Cases and controls were eligible if they were of Hispanic origin. Cases were included if breast cancer diagnosis was in or after January 1, 2004. Controls without any relationship with the cases, without a family history of breast cancer, and without any breast disease were included.

### DNA extraction

Eight milliliters of blood were drawn from each participant of the Col-BCCC; tubes with the anticoagulant EDTA (ethylenediaminetetraacetic acid) were used. Genomic DNA was extracted using a salting-out method.^15^ DNA samples were available from all study participants.

### Selection of tag SNPs

The data set in HapMap (CEU population) and 1000 Genomes Project (CLM population) were compared; SNPs with minor allele frequency (MAF) ⩾5% and *r*^2^ ⩾0.8 with at least 1 tag SNP were selected. The comparison revealed that the *DEAR1* linkage patterns differ between HapMap’s CEU population (3 tag SNPs capture 26 alleles) and the CLM population from the1000 Genomes Project (14 tag SNPs capture 64 alleles). As a consequence, we decided to proceed with data from the 1000 Genomes Project, because the CLM population is expected to be more genetically related to study population under investigation. The tagger program (http://www.broad.mit.edu/mpg/tagger/) was used to select SNPs using pairwise tagging methods.

According to the 1000 Genomes Project (phase 3), the *DEAR1* gene spans the region from position 33611003 to 33647660 on chromosome 1 (GRCh37). Based on genotype data from the CLM population (94 individuals), the *DEAR1* gene region and a 5-kbp putative promoter region (position 33611003 to 33652660) comprise 230 SNPs (all 4 transcripts). Of these 230 SNPs, there were 65 SNPs with an MAF ⩾5%. Eight tag SNPs captured 100% of the alleles of these 65 SNPs at a correlation of *r*^2^ ⩾0.8. The selected tag SNPs are shown in Supplemental Table S4. From a previous tagging SNP approach, genotypes were obtained for 1 SNP in tagging group 1 (rs59983645), for 2 SNPs in tagging group 2 (rs584298 and rs628466), and for rs2927970. Genotypes for rs599167 in tagging group 3 were obtained using the KASP assay. Four SNPs (rs673894, rs35622844, rs10798929, and rs2927966) were not genotyped. In total, 4 of 65 SNPs capture 93.8% (61/65) of the genetic variation in the investigated region considering an MAF ⩾5% and *r*^2^ ⩾0.8.

### Genotyping

Genotyping of rs584298, rs2927970, and rs59983645 was performed by MALDI-TOF (Matrix-Associated Laser Desorption Ionization Time of Flight) mass spectrometry using Sequenom’s MassArray iPLEX system (Agena Bioscience, San Diego, California) and the iPLEX Gold Chemistry. Post-run data were analyzed using Sequenom’s Typer Analyzer software version 4.0.20 (http://agenabio.com/products/massarray-system/).

Genotyping of rs599167 was performed using a KASP genotyping (Kompetitive Allele Specific PCR (polymerase chain reaction)) assay, and the post-run data were analyzed using the SNPviewer software version 4.0.0.0 (http://www.lgcgroup.com/products/kasp-genotyping-chemistry/overview/#.VhY0UEa6KSo). All assay plating was performed on 384-well plates. Primers for each tag SNP were designed using Assay Design Suite software from Agena Bioscience.

### QC guidelines

The QC (quality control) criteria were in accordance with the BCAC (Breast Cancer Association Consortium) and CIMBA consortium (the Consortium of Investigators of Modifiers of BRCA1/2). The QC for samples analyzed by MALDI-TOF includes 5.8% of duplicate samples and at least 95% of the concordance rate. The QCs for samples analyzed by KASP Genotyping Assay included 6.0% of duplicate samples (61 cases and 61 controls). At least 5% duplication with ⩾98% concordance was required.

Four non-DNA controls (NTC—no template control) were included per 384-well plate. A minimum of ⩾2 NTCs was used per plate. DNA samples that failed for >2 of the 4 analyzed SNPs were excluded (8 samples). After exclusion of these samples, the call rate for SNPs was rs584298: 98.5%; rs2927970: 98.7%; rs59983645: 98.3%; rs599167: 95.6%. A minimum call rate of ⩾95% was required. The sample had no significant deviation from HWE (Hardy-Weinberg equilibrium); the HWE analysis data are shown in Supplemental Tables S5 to S8.

### Statistical methods

Differences between breast cancer cases and controls in the frequencies of general characteristics, such as family history, number of childbirths, breastfeeding experience, OC use, BMI, smoking, and age at first full-term pregnancy, were tested using the χ^2^ test.

For each SNP, deviation from HWE was assessed by a χ^2^ with 1 degree of freedom (1 *df*). Associations between genetic variables and breast cancer risk model adjusted for 6 potential epidemiological breast cancer risk factors (age, menopausal status, family history of breast or ovarian cancer, use of OCs, postmenopausal hormone therapy, BMI, and smoking) were analyzed by conditional logistic regression with odds ratios (ORs) and 95% confidence intervals (CIs). All variables were included into the complete model, and to determine which terms were significant, a hierarchical backward elimination approach was carried out. The variables not statistically significant during this procedure were evaluated as possible confounding factors. To select the best model, the Bayesian information criterion and the Akaike information criterion were used. The associations between *DEAR1* genotypes and 7 clinical and histopathological tumor characteristics (histology, histological grade, ER status, PR status, and HER2 status) of breast cancer cases were analyzed by multinomial logistic regression with relative risk ratio and 95% CIs. All tests were 2-sided. Multicollinearity between independent variables was evaluated. All the statistical analyses were performed using Stata software version 15.^16^ Significance was settled at a *P* = .05.

A multivariate analysis of survival, using Kaplan-Meier approach, was conducted, with right censored observations. The survival rates are presented with 95% CIs.

## Results

### Subject characteristics

The ages of the case and control groups were similar with a mean age of 52 years for the interviewees; the case group has a mean age of diagnosis of 49.75 years. Case and control groups show principally a tendency to overweight; no significant statistical differences (*P* > .005) were found. Significant differences between case and control groups are evident for the following variables, smoking habit (*P* = .000388), family history of breast cancer (*P* = .00001), use of OCs (*P* = .0291) and use of hormone replacement therapy (HRT) (*P* = .00001), parity (*P* = .0128), and breastfeeding (*P* = .0265); *BRCA1* or *BRCA2* mutations also show differences between case and control groups (*P* < .0001). The frequency of the variables for case and control groups is shown in Tables 1 and 2.

**Table 1.** Characteristics for cases and controls: age and BMI.

Variable	Sample	No. of observations	Missing data	Media	SE	95% confidence interval	Range	Absolute frequency		Relative frequency (%)	
								Controls	Cases	Controls	Cases
Age of diagnosis	Cases	1019	3 (0.29%)	49.75	0.367	49.03-50.4	<40	–	177		17.35
							40-59	–	641		62.84
							>60	–	201		19.70
Age of interview	Cases	1022	0 (0.00%)	52.04	0.896	51.98-52.09	<40	174	147	17	14.28
							40-59	650	650	63.5	63.6
	Controls	1023		49.95	0.36	49.93-49.97	>60	199	225	19.35	22.01
BMI	Cases	973	49 (4.79%)	25.24	0.128	24.99-25.49	<18.5	12	16	1.17	1.56
							18.5-24.9	432	478	42.22	46.77
	Controls	958	64 (6.25%)	25.74	0.131	25.73-25.75	25-29.9	388	366	37.92	35.81
							> 30	127	113	12.41	11.05

BMI, body mass index.

Controls and cases were matched by age +2 years. Age of diagnosis: age at diagnosis for cases. Age of interview: Age at interview/questionnaire for controls and cases. BMI at interview/questionnaire in kg: underweight < 18.5; normal weight = 18.5-24.9; overweight = 25-29.9; obesity > 30.

**Table 2.** Risk factors for breast cancer in cases and controls.

Risk factors	Cases	Missing data cases	Controls	Missing data controls
Smoking				
Never	684 (67%)	22 (2.15%)	775 (76%)	5 (0.48%)
Past	251 (24.5%)		186 (18.18%)	
Current	65 (6.3%)		57 (5.6%)	
Menopausal status				
Pre/peri	286 (28%)	15 (1.46%)	472 (46.1%)	11 (1.075%)
Postmenopausal	721 (70.5%)		540 (53%)	
Family history				
Yes	248 (24.3%)	6 (0.58%)	79 (7.8%)	23 (2.24%)
No	768 (75.1%)		921 (90%)	
Use of oral contraceptives				
Yes	310 (30.3%)	25 (2.44%)	270 (26.4%)	11 (1.075%)
No	687 (67.22)		742 (73%)	
Use of hormone replacement therapy				
Yes	109 (10.7%)	24 (2.34%)	44 (4.30%)	38 (3.71%)
No	889 (87%)		971 (95%)	
Parity				
Yes	858 (84%)	6 (0.58%)	894 (87.4%)	11 (1.075%)
No	158 (15.5%)		118 (11.5%)	
Age of first full-term pregnancy				
<30	699 (68.4%)	178 (17.4%)	780 (76.2%)	151 (14.7%)
>30	145 (14.2%)		92 (9%)	
Breast feeding				
Yes	769 (75.2%)	192 (18.78%)	817 (80%)	166 (16.22%)
No	61 (6%)		40 (4%)	
*BRCA1* mutations				
Yes	55 (5.4%)	0 (0.00%)	0	0 (0.00%)
No	949 (92.8%)		1023 (100%)	
*BRCA2* mutations				
Yes	18 (1.76%)	0 (0.00%)	0	0 (0.00%)
No	949 (93%)		1023 (100%)	

BRCA, breast cancer genes.

Epidemiological risk factors, smoking: 0 = never, 1 = past, 2 = current, in the last year before the reference date (year before diagnosis for cases, year before questionnaire for controls). Menopausal status: 1 = pre/peri, 2 = post (postmenopausal: last menstruation more than 12 months before the reference date). Family history: family history of breast cancer in a first degree relative; 0 = no, 1 = yes. Use of oral contraceptive: 0 = never, 1 = ever, and “never use” correspondingly less than 4 months of use; “ever use” is usually defined as at least 4 months of use. Use of hormone replacement therapy: 0 = never, 1 = ever, “never use” correspondingly less than or equal to 3 months of use, “ever use” is usually defined as more than 3 months of use.

### Association between SNPs and breast cancer risk adjusted for 6 potential epidemiological risk factors

Four tag SNPs in the *DEAR1* gene were selected for genotyping: rs59983645, rs584298, rs2927970, and rs62846 representing 68 common SNPs into the region spanning from 33611003 to 33652660 in chromosome 1. The 4 SNPs selected were genotyped in the total of case and control groups; they were analyzed and cataloged in the VariantGPS software in order to know whether they were in any regulatory region; one of the imputed SNP was found in the miRNA site binding in silico (data not shown). The genotype distributions of all SNPs in controls did not deviate from HWE. The results showed association of tag SNP rs584298 with breast cancer risk genotype AG and GG (*P* = .048 and *P* = .004 respectively) after adjusting for age, family history, BMI, menopausal status, replacement hormonal therapy, and breastfeeding. The 3 other tag SNPs individually and adjusted for the epidemiological risk variables show association, but in the complete model including all 4 tag SNPs did not show the same result because of the correlation between each other. Furthermore, no statistically significant difference was found. The conditional logistic regression analysis for the full model and the reduced model with epidemiological risk variables is shown in Table 3.

**Table 3.** Genotype frequencies, OR, and probabilities.

Tag SNPs	Genotypes	Frequencies patients		Full model			Reduced model		
		Cases	Controls	OR	95% CI	*P* value	OR^a^	95% CI^a^	*P* value^a^
rs584298	AA	498	541						
	GG	74	63	2.24	0.88-5.70	.09	2.57	1.35-4.90	.004
	AG	371	360	1.44	0.86-2.41	.164	1.41	1.00-2.00	.048
rs2927970	CC	149	132						
	TT	366	392	0.9	0.44-1.88	.798			
	TC	425	440	0.85	0.44-1.62	.624			
Risk variables									
Age				7.15	5.21-9.82	.000	7.28	5.32-9.97	.000
Menopausal status				2.25	1.36-3.73	.002	2.28	1.39-3.74	.001
BMI				0.99	0.997-0.999	.004	0.99	0.997-0.999	.004
Family history				4.31	2.68-6.92	.000	4.54	2.84-7.26	.000
Parous				0.65	0.28-1.50	.323			
Smoking				0.98	0.48-2.00	.967			
Breast feeding				1.07	0.52-2.20	.847	1.41	0.93-2.15	.100
OC ever				1	0.701-1.43	.993			
HRT ever				2.27	1.16-4.43	.150	2.4	1.25-4.58	.008

BMI, body mass index; CI, confidence interval; HRT, hormone replacement therapy; OC, oral contraceptive; OR, odds ratio; SNP, single nucleotide polymorphism.

a Adjusted analysis by age, menopausal status, HRT, family history, breastfeeding, and BMI; *P* < 0.05. (These variables were included due to the significant risk association for breast cancer in different populations.)

An evaluation of multicollinearity between risk factors and genotypes was performed. Risk variables age and menopausal status show high correlation but not collinearity. Tag SNPs rs599167 and 59983645 show collinearity; for this reason, these 2 SNPs were not included in the final conditional logistic regression model; no other SNPs showed significance level for entry into the model.

### Tag SNPs association with histopathological tumor characteristics

In order to evaluate whether genotypes in the *DEAR1* gene were associated with some specific tumoral characteristics, a multinomial logistic regression was carried out between *DEAR1* genotypes and 5 clinical and histopathological tumor characteristics (histology, histological grade, ER status, PR status, and HER2 status) (data are shown in Tables 4 and 5). The results show association between tag SNPs rs584298 genotype AA with HER2 receptor expression (*P* = .048), and genotype AG with PR expression (*P* = .015); therefore, if 1 patient has HER2 expression, the relative risk of having the genotype AA over GG (referent group) would increase the risk by a factor of 3.48 times when the other variables in the model are held constant. The association with PR status will also increase: if a patient has a PR expression, the relative risk to have the genotype AG over GG (referent group) would be expected to increase by a factor of 2.78 times when the other variables in the model are held constant (data are shown in Table 6).

**Table 4.** Description of histopathological variables.

Histopathological variables	Frequency	%
Tumor type		
Invasive^a^	731	71.52
In situ	137	13.40
Unknown	154	15.06
ER status		
Negative	153	14.97
Positive	541	52.93
Unknown	328	32.09
PR status		
Negative	191	18.68
Positive	498	48.72
Unknown	333	32.58
HER2 status		
Negative	419	40.99
Positive	133	13.01
Unknown	470	45.98

ER, estrogen receptor; HER2, human epidermal growth factor receptor 2; PR, progesterone receptor.

ER-positive: Breast cancers that have ERs are called ER-positive (or ER +) cancers; PR-positive: Breast cancers with PRs are called PR-positive (or PR +) cancers. Unknown: missing data.

a Invasive included lobular, ductal.

**Table 5.** Description of variables of tumor size and number of nodes.

Histopathological variables	Media	SE	95% CI	Total observations	Missing data
Tumor size^a^	23.30	0.655	22.01-24.59	532	490 (47.94%)
Node positive	1.95	0.172	1.61-2.28	602	420 (41.09%)

CI, confidence interval.

a Tumor size is given in millimeters; Node positive: number of positive nodules in the pathology report.

**Table 6.** Tag SNP association between rs584298 and PR, HER2 status.

Tag SNP	RRR	SE	z	*P* > |z|	95% CI
rs584298					
A					
PR Status	1.758	0.706	1.40	.161	0.79-3.86
HER2 status	3.48	2.20	1.98	.048	1.00-12.01
AG					
PR status	2.78	1.17	2.43	.015	1.22-6.36
HER2 status	3.10	2.00	1.76	.078	0.879-10.99
G	Base outcome				

CI, confidence interval; HER2, human epidermal growth factor receptor 2; PR, progesterone receptor; RRR, relative risk ratio; SNP, single nucleotide polymorphism.

Association results for the Tag SNP rs584298 genotype AA and AG. Adjusted for tumor size and node status.

### Survival analysis

For survival analysis, the endpoints were as follows: (1) time for recurrence: defined as the time from diagnosis to a random documentation of a breast event; any local, regional, or distant recurrence of breast cancer; or a contralateral breast cancer; (2) breast cancer–specific survival (BCSS): defined as the time from diagnosis to death from breast cancer; (3) overall-free survival (OSS): estimated as the time from registration to death of any cause.

**Figure 1.**
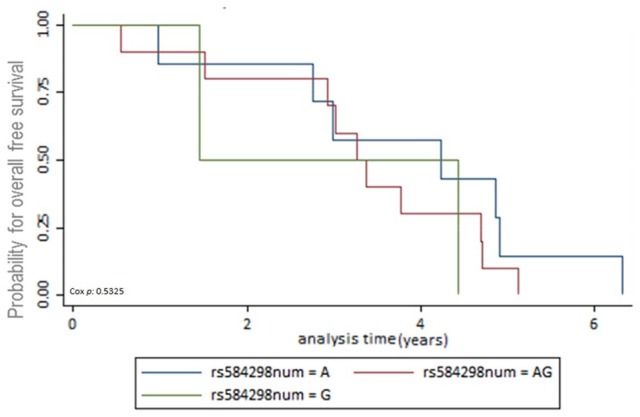
Kaplan-Meier survival estimates for overall-free survival (OSS). Genotypes for rs584298 resulted in nonsignificant correlation between the genotypes AA, GG and AG and overall-free survival (*P =* .5325).

**Figure 2.**
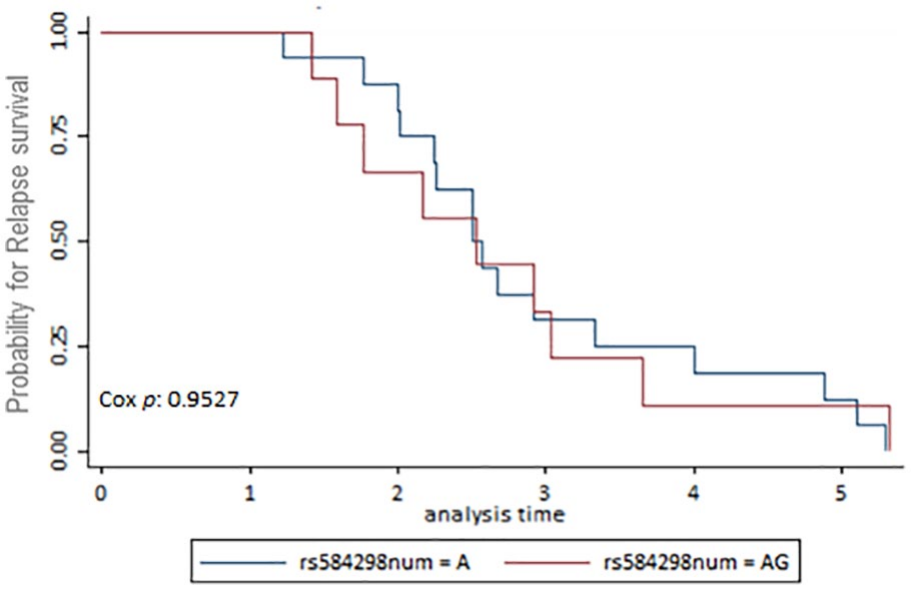
Kaplan-Meier survival estimates for relapse. Genotypes for rs584298 resulted in nonsignificant correlation between the genotypes AA and AG and relapse-free survival (*P* = .952).

**Figure 3.**
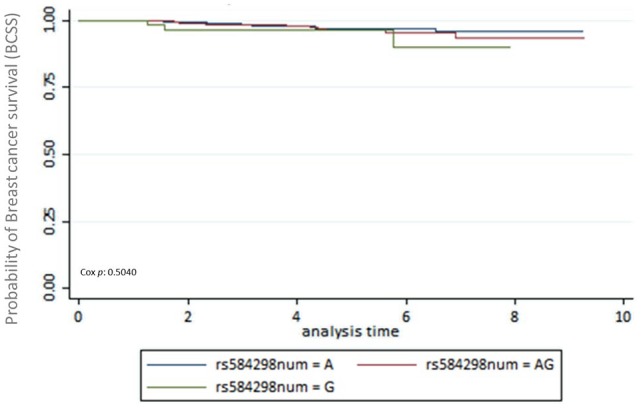
Kaplan-Meier survival estimates for breast cancer–specific survival (BCSS). Genotypes for rs584298 resulted in nonsignificant correlation between the genotypes AA, GG and AG and specific breast cancer–free survival (*P* = .5040).

The first end-point, time to recurrence, evidenced that 42 patients had contralateral breast cancer or locoregional disease. From these 42 patients, 31 were censored and 11 died. Data available for BCSS and OSS analyses were as follows: 77 (7.5%) patients died from the total of 1022. After 5-year follow-up, from these 75 patients, 26 (33.7%) patients died due to breast cancer, 18 (23.37%) died for a cause different to breast cancer, and 33 (42.85%) died for unknown cause. A total of 318 (31.11%) patients had a follow-up missed out from the total. The number of patients alive until the date of the last follow-up was 627 (61.35%). The multiple analyses of survival were conducted using the Kaplan-Meier survival method. There was no evidence of association between SNPs in the *DEAR1* region and BCSS, OSS, and time to recurrence. Survival Kaplan-Meier data are shown in Figures 1 to 3 and Supplemental Tables S1 to S3.

## Discussion

Most cases and controls were in postmenopausal status and were overweight with a BMI > 25. Those both elements are considered as a risk factor for breast cancer.^17^,^18^ Cases were mainly diagnosed in their fourth and fifth decades, and the majority shared characteristics considered as protective factors for the disease such as parity, age of women at birth of first child (less than 30 years old), breastfeeding, and no use of HRT or OCs. Most of the established breast cancer risk factors evaluated among Colombian women in the current study had strong associations with breast cancer.

The *DEAR1* gene located in the region 1p35.1 encodes for a member of the tripartite motif (TRIM) protein, one of the subfamilies of the RING type E3 ubiquitin ligases, which is associated with the development of breast cancer and invasion.^3^ Functional cell line mutational analysis provides evidence that *DEAR1* is a key regulator of acinar morphogenesis in the mammary gland and an independent predictor of local recurrence-free survival. The association between *DEAR1* and breast cancer suggests that *DEAR1* polymorphisms could be a susceptibility factor for this disease.^3^ Besides the obvious role as a key regulator of acinar morphogenesis, *DEAR1* gene has recently been considered a regulator of p27 stability, and an inhibitor of cyclin-dependent kinases. Decreased TRIM62 expression by RNA interference in cancer HER2 + cell lines not only induces cell cycle arrest but also causes the complete relocation of p27 to the nucleus.^19^ The loss of function of *DEAR1* gene in the presence of TGF-β results in a failure in the acinar morphogenesis, upregulation of EMT markers, resistance to anoikis, migration, and invasion; therefore, *DEAR1* is a master regulator of EMT.^3^ The loss of *DEAR1* expression has also been considered an adverse prognostic factor in acute myeloid leukemia.^20^ In nonsmall cell lung cancer lesions, the loss of TRIM62 levels is lower during disease progression, which has been associated with poor clinical outcomes.^21^ The effect of this gene on breast cancer, as determined by functional analysis, increases the probability of one or many variants being associated with breast cancer risk. Until now, no reports about variants in *DEAR1* associated with breast cancer risk adjusted for different epidemiological risk factors had been conducted and it is important to highlight the role of interactions between genotype, risk factors, and breast cancer risk, but the current approaches to identify such interactions are scarce, and new methodologies should be developed. This is the first report of *DEAR1* variants associated with breast cancer risk; further studies in other populations are needed to confirm these slight associations between breast cancer risk and rs584298 as well as PR status and HER2 status.

Four SNPs (rs584298, rs2927970, rs59983645, rs599167) in the *DEAR1* gene were genotyped. The association with breast cancer risk was adjusted with epidemiological risk factors in a sample of the Colombian population. After adjusting for the covariates of epidemiological risk, the association with the tag SNPs rs584298 and breast cancer risk was found. This SNP despite of the similar frequency in different populations has no report of clinical significance.^4^ The rs584298 tagged an SNP (rs689187) located in 3′-UTR region of TRIM62, an miRNA binding site therefore probably causal variant associated with breast cancer risk. These 2 variants have a high *r*^2^ and *D*′ values, showing that they are coinherited. Further analyses are needed to confirm whether rs689187 is affecting the miRNA binding site and whether this could be a causal variant associated with breast cancer progression. Other replication studies are required to confirm that these variants could be a prognostic factor for patients with in situ breast cancer.^22^ Some genome-wide association studies have evaluated interactions between identified genetic variants and traditional breast cancer risk factors; none found significant interactions after correction for the number of comparisons made, and all were conducted among predominantly European-ancestry populations.^23^ In this case, probably the lack of strong associations should be due principally to the size and diversity of the populations evaluated, the number of SNPs tagged, and the missing data for each variable. Additional studies in other populations should be conducted.

The allele A of rs584298 was associated with the expression of HER2 receptor; therefore, it would be expected that rs689187 would be the causal variant associated; several studies had shown the importance of SNPs or miRNA binding sites polymorphism, mainly SNPs within the 3′-UTR gene region which may influence and regulate the posttranscription modulation on gene expression and in this manner confer susceptibility to the disease. HER2 overexpression has an important prevalence, nearly to ~22% in breast cancer; the upregulation of HER2 is associated with different histological characteristics of the tumor, metastasis and invasiveness, angiogenesis, and a poor overall prognosis.^24^ Several miRNA have been associated with overexpression of HER2.^25^ Also, the genotype AG of rs584298 in TRIM62 shows association with expression of PR status, possibly the causal variant could be the SNP in the miRNA binding site rs689187 affecting the expression of PR. Further studies are needed to identify the possible miRNA associated with the 3-UTR region in TRIM62 and the pathway involved in the HER2 dysregulation to establish it as a possible prognostic marker in breast cancer.

Survival analysis show more than 90% of censored data with more than 30% unknown data, making it difficult to perform Kaplan-Meier analysis and probably this is one of the reasons for nonassociation.^26^

## Conclusions

SNP rs584298 in *DEAR1* showed association with positive HER2 and PR receptors breast cancer risk in a sample of Colombian population; therefore, this information could be relevant for therapeutic decisions and prognosis. This SNP should be tested in other populations to confirm the association in a larger sample. In the future, those SNP could be considered as part of cancer risk and prognosis panels. It is crucial to perform SNP validation (rs689187) of the SNP in the miRNA binding site with functional analysis to determine the SNP targets.

## Limitations of the Study

Some limitations in the study were related principally with the tumor pathological information and follow-up. Shortly after the patients were diagnosed, they were referred to the study; therefore, they did not have all the pathological information, making it difficult to fill all the required information.

## Supplemental Material

SUPPLEMENTAL_INFORMATION_DEAR1_xyz3051994c94f72 – Supplemental material for Association of *DEAR1* Tagging Single Nucleotide Polymorphisms With Breast Cancer in a Sample of Colombian Population: A Case Control Study Click here for additional data file. Supplemental material, SUPPLEMENTAL_INFORMATION_DEAR1_xyz3051994c94f72 for Association of *DEAR1* Tagging Single Nucleotide Polymorphisms With Breast Cancer in a Sample of Colombian Population: A Case Control Study by Angela P Beltrán, Edgar Benitez, Martin Rondon, Yeimy V Ariza, Fabio A Aristizabal and Ignacio Briceño in Breast Cancer: Basic and Clinical Research

## References

[1] Lott ST Chen N Chandler DS, et al DEAR1 is a dominant regulator of acinar morphogenesis and an independent predictor of local recurrence-free survival in early-onset breast cancer. PLoS Med. 2009;6:e1000068. doi:10.1371/journal.pmed.1000068. PMC2673042 19536326

[2] Ragnarsson G Eiriksdottir G Johannsdottir JT Jonasson JG Egilsson V Ingvarsson S. Loss of heterozygosity at chromosome 1p in different solid human tumours: association with survival. Br J Cancer. 1999;79:1468-1474. doi:10.1038/sj.bjc.6690234. 10188892 PMC2362732

[3] Chen N Balasenthil S Reuther J, et al DEAR1 is a chromosome 1p35 tumor suppressor and master regulator of TGF-β-driven epithelial-mesenchymal transition. Cancer Discov. 2013;3:1172-1189. doi:10.1158/2159-8290.CD-12-0499. 23838884 PMC4107927

[4] Auton A Brooks LD Durbin RM, et al A global reference for human genetic variation. Nature. 2015;526:68-74. doi:10.1038/nature15393. 26432245 PMC4750478

[5] Frazer KA Ballinger DG Cox DR, et al A second generation human haplotype map of over 3.1 million SNPs. Nature. 2007;449:851-861. doi:10.1038/nature06258. 17943122 PMC2689609

[6] Foulkes WD. Inherited susceptibility to common cancers. N Engl J Med. 2008;359:2143-2153. doi:10.1056/NEJMra0802968. 19005198

[7] Metcalfe K Lubinski J Lynch HT, et al Family history of cancer and cancer risks in women with BRCA1 or BRCA2 mutations. J Natl Cancer Inst. 2010;102:1874-1878. doi:10.1093/jnci/djq443. 21098759

[8] Thompson D Easton D. The genetic epidemiology of breast cancer genes. J Mammary Gland Biol Neoplasia. 2004;9:221-236. doi:10.1023/B:JOMG.0000048770.90334.3b. 15557796

[9] Pharoah PDP Dunning AM Ponder BAJ Easton DF Association studies for finding cancer-susceptibility genetic variants. Nat Rev Cancer. 2004;4:850-860. 15516958 10.1038/nrc1476

[10] Dai J Hu Z Jiang Y, et al Breast cancer risk assessment with five independent genetic variants and two risk factors in Chinese women. Breast Cancer Res. 2012;14:R17. doi:10.1186/bcr3101. 22269215 PMC3496134

[11] Harlid S Ivarsson MI Butt S, et al Combined effect of low-penetrant SNPs on breast cancer risk. Br J Cancer. 2012;106:389-396. doi:10.1038/bjc.2011.461. 22045194 PMC3261688

[12] Jostins L Barrett JC. Genetic risk prediction in complex disease. Hum Mol Genet. 2011;20:R182-R188. doi:10.1093/hmg/ddr378. 21873261 PMC3179379

[13] Torres D Bermejo JL Rashid MU, et al Prevalence and penetrance of BRCA1 and BRCA2 germline mutations in Colombian breast cancer patients. Sci Rep. 2017;7:4713. doi:10.1038/s41598-017-05056-y. 28680148 PMC5498630

[14] Pineros M Cendales R Murillo R Wiesner C Tovar S. [Pap test coverage and related factors in Colombia, 2005]. Rev Salud Publica (Bogota). 2007;9:327-341. 18026598 10.1590/s0124-00642007000300002

[15] Miller SA Dykes DD Polesky HF. A simple salting out procedure for extracting DNA from human nucleated cells. Nucleic Acids Res. 1988;16:1215. 3344216 10.1093/nar/16.3.1215 PMC334765

[16] StataCorp. Statistical Software: Release 15. College Station, TX: StataCorp LLC, 2017.

[17] Gravena AAF Romeiro Lopes TC Demitto MO, et al The obesity and the risk of breast cancer among pre and postmenopausal women. Asian Pac J Cancer Prev. 2018;19:2429-2436. doi:10.22034/APJCP.2018.19.9.2429. 30255696 PMC6249449

[18] Neuhouser ML Aragaki AK Prentice RL, et al Overweight, obesity, and postmenopausal invasive breast cancer risk: a secondary analysis of the women’s health initiative randomized clinical trials. JAMA Oncol. 2015;1:611-621. doi:10.1001/jamaoncol.2015.1546. 26182172 PMC5070941

[19] Faltermeier CMEE Roberts JM. Abstract A64: mechanistic insights into the misregulation of p27 in HER2 - positive breast cancers. Paper presented at the AACR-NCI-EORTC International Conference: Molecular Targets and Cancer Therapeutics; November 15-19, 2009, pp. 15-19. https://mct.aacrjournals.org/content/8/12_Supplement/A64.

[20] Quintas-Cardama A Zhang N Qiu YH, et al Loss of TRIM62 expression is an independent adverse prognostic factor in acute myeloid leukemia. Clin Lymphoma Myeloma Leuk. 2015;15:115-127. doi:10.1016/j.clml.2014.07.011. 25248926 PMC4560255

[21] Quintas-Cardama A Post SM Solis LM, et al Loss of the novel tumour suppressor and polarity gene Trim62 (Dear1) synergizes with oncogenic Ras in invasive lung cancer. J Pathol. 2014;234:108-119. doi:10.1002/path.4385. 24890125 PMC4138305

[22] Tonini G D’Onofrio L Dell’Aquila E Pezzuto A. New molecular insights in tobacco-induced lung cancer. Future Oncol. 2013;9:649-655. doi:10.2217/fon.13.32. 23647294

[23] Campa D Kaaks R Le Marchand L, et al Interactions between genetic variants and breast cancer risk factors in the breast and prostate cancer cohort consortium. J Natl Cancer Inst. 2011;103:1252-1263. doi:10.1093/jnci/djr265. 21791674 PMC3156803

[24] Ross JS Slodkowska EA Symmans WF Pusztai L Ravdin PM Hortobagyi GN. The HER-2 receptor and breast cancer: ten years of targeted anti-HER-2 therapy and personalized medicine. Oncologist. 2009;14:320-368. doi:10.1634/theoncologist.2008-0230. 19346299

[25] Lowery AJ Miller N Devaney A, et al MicroRNA signatures predict oestrogen receptor, progesterone receptor and HER2/neu receptor status in breast cancer. Breast Cancer Res. 2009;11:R27. doi:10.1186/bcr2257. 19432961 PMC2716495

[26] De Bakker PI Yelensky R Pe’er I Gabriel SB Daly MJ Altshuler D. Efficiency and power in genetic association studies. Nat Genet. 2005;37:1217-1223. doi:10.1038/ng1669. 16244653

