# Supplementary material for: Association of DEAR1 Tagging Single Nucleotide Polymorphisms With Breast Cancer in a Sample of Colombian Population: A Case Control Study
Source: Breast Cancer (Auckl). 2020 Apr 20;14:1178223420904939. doi: 10.1177/1178223420904939 (PMC7172001; doi:10.1177/1178223420904939)
Supplement: SUPPLEMENTAL_INFORMATION_DEAR1_xyz3051994c94f72 – Supplemental material for Association of DEAR1 Tagging Single Nucleotide Polymorphisms With Breast Cancer in a Sample of Colombian Population: A Case Control Study [file SUPPLEMENTAL_INFORMATION_DEAR1_xyz3051994c94f72.pdf]

Table SI 1. Data to survival analysis for BCSS

```
. stcox i. rs584298num

         failure _d:  BrDeath (BREAST CANCER DEATH)
   analysis time _t:  YearsToStatus

Iteration 0:   log likelihood = -156.04171
Iteration 1:   log likelihood =  -155.3893
Iteration 2:   log likelihood = -155.35653
Iteration 3:   log likelihood = -155.35644
Refining estimates:
Iteration 0:   log likelihood = -155.35644

Cox regression -- Breslow method for ties

No. of subjects =          701                  Number of obs    =         701
No. of failures =           25
Time at risk    =  3708.060212
                                                LR chi2(2)       =        1.37
Log likelihood  =   -155.35644                  Prob > chi2      =      0.5040

------------------------------------------------------------------------------
          _t | Haz. Ratio   Std. Err.      z    P>|z|     [95% Conf. Interval]
-------------+----------------------------------------------------------------
 rs584298num |
        AG  |   1.389957    .592845     0.77   0.440     .6024851    3.206684
         G  |   2.088703   1.363178     1.13   0.259     .5812269    7.505986
------------------------------------------------------------------------------
```

Table SI-2.: **Data of survival analysis for OSS**

```
stcox i. rs584298num

         failure _d:  VitalStatus (Overall survival)
   analysis time _t:  YearsToStatus

Iteration 0:   log likelihood = -39.339884
Iteration 1:   log likelihood = -38.719638
Iteration 2:   log likelihood =  -38.70969
Iteration 3:   log likelihood = -38.709682
Refining estimates:
Iteration 0:   log likelihood = -38.709682

Cox regression -- no ties

No. of subjects =           19                  Number of obs    =          19
No. of failures =           19
Time at risk    =   65.8244627
                                                LR chi2(2)       =        1.26
Log likelihood  =   -38.709682                  Prob > chi2      =      0.5325

------------------------------------------------------------------------------
          _t | Haz. Ratio   Std. Err.      z    P>|z|     [95% Conf. Interval]
-------------+----------------------------------------------------------------
 rs584298num |
        AG  |    1.61502   .8537055     0.91   0.365     .5730991    4.551203
```

```
          G  |   2.274046   1.932563     0.97   0.334      .429951    12.02762
------------------------------------------------------------------------------
```

Table SI-3.: **Data of survival analysis for Recurrence**

```
. stcox i. rs584298num

         failure _d:  Locoregional_relapse_cat (recurrence)
   analysis time _t:  years_locoreg_relapse

Iteration 0:   log likelihood = -58.003605
Iteration 1:   log likelihood = -58.001844
Iteration 2:   log likelihood = -58.001844
Refining estimates:
Iteration 0:   log likelihood = -58.001844

Cox regression -- no ties

No. of subjects =           25                     Number of obs    =          25
No. of failures =           25
Time at risk    =  71.67967105
                                                   LR chi2(1)       =        0.00
Log likelihood  =   -58.001844                     Prob > chi2      =      0.9527

------------------------------------------------------------------------------
          _t | Haz. Ratio   Std. Err.      z    P>|z|     [95% Conf. Interval]
-------------+----------------------------------------------------------------
 rs584298num |
         AG  |   .9744651   .4253122    -0.06   0.953     .4142412    2.292341
------------------------------------------------------------------------------
```

Table SI 4. Tag SNPs and alleles captured by the genotyping SNPs

Tag SNP	*MAF	Alleles captured
rs584298	0,217	rs654336, rs685235, rs621539, rs584298, rs599167, rs689187, rs641657, rs607008, rs784773, rs631646
rs59983645	0,15	rs35111341,rs12734992,rs72656238,rs59983645,rs623596,rs3912145,rs12563712,rs12046456,rs4481817,rs57784925,rs3863726,rs3766813,rs35643025,rs71647911,rs79987911,rs2306257,rs17465232,rs12026515,rs7541761, rs75976605, rs35164421, rs72885925
rs2927970	0,367	rs2927970
rs599167	0,21	rs599161,rs631646,rs654336,rs623596,,rs685235,rs641657,rs607008,rs784773,rs621539

- MAF: minor allele frequency
- SNPs in miRNA binding sites

Table SI 5. **Test for deviation from Hardy-Weinberg equilibrium for rs584298**

	Tests for deviation from Hardy-Weinberg equilibrium		Tests for association (C.I.: 95% confidence interval)				
	**Controls**	**Cases**	**allele freq. difference**	**heterozygous**	**homozygous**	**allele positivity**	**Armitage's trend test**
rs584298	n11(AA)=565 (563.82)<br>n12(AG)=380 (382.35)<br>n22 (GG)=66 (64.82)<br>f_a1=0.75 +/- 0.010<br>F=0.00616<br>p=0.844794 (Pearson)<br>p=0.844994 (Llr)<br>p=0.867873 (Exact)	n11(AA)=533 (532.76)<br>n12 (AG)=396 (396.47)<br>n22(GG)=74 (73.76)<br>f_a1=0.73 +/-0.010<br>F=0.00120<br>p=0.969760 (Pearson)<br>p=0.969767 (Llr)<br>p=1.000000 (Exact)	**Risk allele 2**				
			**[1]<->[2]**	**[11]<->[12]**	**[11+]<->[22]**	**[11]<->[12+22]**	**common odds ratio**
			Odds_ratio=1.097<br>C.I.=[0.954-1.263]<br>chi2=1.68<br>p=0.19474 (P)	Odds_ratio=1.105<br>C.I.=[0.919-1.328]<br>chi2=1.13<br>p=0.28863	Odds_ratio=1.189<br>C.I.=[0.836-1.690]<br>chi2=0.92<br>p=0.33621	Odds_ratio=1.117<br>C.I.=[0.937-1.331]<br>chi2=1.53<br>p=0.21616	Odds_ratio=1.095<br>chi2=1.67<br>p=0.19564
			**Risk allele 1**				
			**[2]<->[1]**	**[22]<->[12]**	**[22]<->[11]**	**[11+12]<->[22]**	**common odds ratio**
			Odds_ratio=0.911<br>C.I.=[0.792-1.049]<br>chi2=1.68<br>p=0.19474 (P)	Odds_ratio=0.929<br>C.I.=[0.648-1.333]<br>chi2=0.16<br>p=0.69070	Odds_ratio=0.841<br>C.I.=[0.592-1.197]<br>chi2=0.92<br>p=0.33621	Odds_ratio=0.877<br>C.I.=[0.622-1.237]<br>chi2=0.56<br>p=0.45346	Odds_ratio=0.913<br>chi2=1.67<br>p=0.19564

The tests for association are adapted from Sasieni PD (1997). f a1:Frequency of allele 1 +/- standard deviation; F:Inbreeding coefficient;

p (Pearson):Pearson's goodness-of-fit chi-square (degree of freedom = 1); p (Llr):Log likelihood ratio chi-square (degree of freedom = 1);

p (Exact):Exact test

Table SI 6. **Test for deviation from Hardy-Weinberg equilibrium for rs2927970**

	**Tests for deviation from Hardy-Weinberg equilibrium**		**Tests for association (C.I.: 95% confidence interval)**				
	**Controls**	**Cases**	**allele freq. difference**	**heterozygous**	**homozygous**	**allele positivity**	**Armitage's trend test**
rs2927970	n11 (TT)=410 (403.81)<br>n12(TC)=456 (468.37)<br>n22(CC)=142 (135.81)<br>f_a1=0.63 +/- 0.011<br>F=0.02642<br>p=0.401630 (Pearson)<br>p=0.402215 (Llr)<br>p=0.415962 (Exact)	n11 (TT)=389 (377.53)<br>n12 (TC)=457 (479.94)<br>n22(CC)=164 (152.53)<br>f_a1=0.61 +/- 0.011<br>F=0.04779<br>p=0.128784 (Pearson)<br>p=0.129317 (Llr)<br>p=0.128133 (Exact)			**Risk allele 2**		
			**[1]<->[2]**	**[11]<->[12]**	**[11+]<->[22]**	**[11]<->[12+22]**	**common odds ratio**
			Odds_ratio=1.096<br>C.I.=[0.965-1.245]<br>chi2=1.99<br>p=0.15799 (P)	Odds_ratio=1.056<br>C.I.=[0.874-1.277]<br>chi2=0.32<br>p=0.57195	Odds_ratio=1.217<br>C.I.=[0.935-1.585]<br>chi2=2.13<br>p=0.14418	Odds_ratio=1.095<br>C.I.=[0.916-1.308]<br>chi2=0.98<br>p=0.32123	Odds_ratio=1.097<br>chi2=1.92<br>p=0.16576
					**Risk allele 1**		
			**[2]<->[1]**	**[22]<->[12]**	**[22]<->[11]**	**[11+12]<->[22]**	**common odds ratio**
			Odds_ratio=0.912<br>C.I.=[0.803-1.036]<br>chi2=1.99<br>p=0.15799 (P)	Odds_ratio=0.868<br>C.I.=[0.669-1.125]<br>chi2=1.15<br>p=0.28371	Odds_ratio=0.822<br>C.I.=[0.631-1.070]<br>chi2=2.13<br>p=0.14418	Odds_ratio=0.846<br>C.I.=[0.663-1.079]<br>chi2=1.81<br>p=0.17811	Odds_ratio=0.912<br>chi2=1.92<br>p=0.16576

The tests for association are adapted from Sasieni PD (1997). f a1:Frequency of allele 1 +/- standard deviation; F:Inbreeding coefficient;

p (Pearson):Pearson's goodness-of-fit chi-square (degree of freedom = 1); p (Llr):Log likelihood ratio chi-square (degree of freedom = 1);

p (Exact):Exact test

Table SI 7. **Test for deviation from Hardy-Weinberg equilibrium for rs59983645**

	Tests for deviation from Hardy-Weinberg equilibrium		Tests for association (C.I.: 95% confidence interval)				
	**Controls**	**Cases**	**allele freq. difference**	**heterozygous**	**homozygous**	**allele positivity**	**Armitage's trend test**
rs59983645	n11(CC)=630 (621.84)<br>n12(TC)=325 (341.32)<br>n22(TT)=55 (46.84)<br>f_a1=0.78 +/- 0.009<br>F=0.04783<br>p=0.128526 (Pearson)<br>p=0.133925 (Llr)<br>p=0.135954 (Exact)	n11(CC)=596 (594.44)<br>n12(TC)=350 (353.12)<br>n22(TT)=54 (52.44)<br>f_a1=0.77 +/- 0.009<br>F=0.00883<br>p=0.780071 (Pearson)<br>p=0.780587 (Llr)<br>p=0.788221 (Exact)	**Risk allele 2**				
			**[1]<->[2]**	**[11]<->[12]**	**[11+]<->[22]**	**[11]<->[12+22]**	**common odds ratio**
			Odds_ratio=1.082<br>C.I.=[0.933-1.256]<br>chi2=1.08<br>p=0.29776 (P)	Odds_ratio=1.138<br>C.I.=[0.943-1.374]<br>chi2=1.83<br>p=0.17657	Odds_ratio=1.038<br>C.I.=[0.701-1.536]<br>chi2=0.03<br>p=0.85265	Odds_ratio=1.124<br>C.I.=[0.939-1.345]<br>chi2=1.63<br>p=0.20200	Odds_ratio=1.061<br>chi2=1.05<br>p=0.30449
			**Risk allele 1**				
			**[2]<->[1]**	**[22]<->[12]**	**[22]<->[11]**	**[11+12]<->[22]**	**common odds ratio**
			Odds_ratio=0.924<br>C.I.=[0.796-1.072]<br>chi2=1.08<br>p=0.29776 (P)	Odds_ratio=1.097<br>C.I.=[0.732-1.644]<br>chi2=0.20<br>p=0.65424	Odds_ratio=0.964<br>C.I.=[0.651-1.426]<br>chi2=0.03<br>p=0.85265	Odds_ratio=1.009<br>C.I.=[0.686-1.484]<br>chi2=0.00<br>p=0.96404	Odds_ratio=0.942<br>chi2=1.05<br>p=0.30449

The tests for association are adapted from Sasieni PD (1997). f a1:Frequency of allele 1 +/- standard deviation; F:Inbreeding coefficient;

p (Pearson):Pearson's goodness-of-fit chi-square (degree of freedom = 1); p (Llr):Log likelihood ratio chi-square (degree of freedom = 1);

p (Exact):Exact test

Table SI 8. **Test for deviation from Hardy-Weinberg equilibrium for rs599167**

	**Tests for deviation from Hardy-Weinberg equilibrium**		**Tests for association (C.I.: 95% confidence interval)**				
	**Controls**	**Cases**	**allele freq. difference**	**heterozygous**	**homozygous**	**allele positivity**	**Armitage's trend test**
rs599167	n11(TT)=583 (577.63)<br>n12(TA)=338 (348.73)<br>n22(AA)=58 (52.63)<br>f_a1=0.77 +/-0.010<br>F=0.03077<br>p=0.335627 (Pearson)<br>p=0.339410 (Llr)<br>p=0.324788 (Exact)	n11 (TT)=571 (563.53)<br>n12(TA)=342 (356.95)<br>n22(AA)=64 (56.53)<br>f_a1=0.76 +/-0.010<br>F=0.04188<br>p=0.190496 (Pearson)<br>p=0.194743 (Llr)<br>p=0.189480 (Exact)			**Risk allele 2**		
			**[1]<->[2]**	**[11]<->[12]**	**[11+]<->[22]**	**[11]<->[12+22]**	**common odds ratio**
			Odds_ratio=1.049<br>C.I.=[0.905-1.216]<br>chi2=0.41<br>p=0.52358 (P)	Odds_ratio=1.033<br>C.I.=[0.855-1.249]<br>chi2=0.11<br>p=0.73629	Odds_ratio=1.127<br>C.I.=[0.775-1.637]<br>chi2=0.39<br>p=0.53143	Odds_ratio=1.047<br>C.I.=[0.874-1.254]<br>chi2=0.25<br>p=0.61889	Odds_ratio=1.052<br>chi2=0.39<br>p=0.53098
					**Risk allele 1**		
			**[2]<->[1]**	**[22]<->[12]**	**[22]<->[11]**	**[11+12]<->[22]**	**common odds ratio**
			Odds_ratio=0.953<br>C.I.=[0.822-1.105]<br>chi2=0.41<br>p=0.52358 (P)	Odds_ratio=0.917<br>C.I.=[0.623-1.349]<br>chi2=0.19<br>p=0.65965	Odds_ratio=0.888<br>C.I.=[0.611-1.290]<br>chi2=0.39<br>p=0.53143	Odds_ratio=0.898<br>C.I.=[0.622-1.297]<br>chi2=0.33<br>p=0.56688	Odds_ratio=0.951<br>chi2=0.39<br>p=0.53098

The tests for association are adapted from Sasieni PD (1997). f a1:Frequency of allele 1 +/- standard deviation; F:Inbreeding coefficient;

p (Pearson):Pearson's goodness-of-fit chi-square (degree of freedom = 1); p (Llr):Log likelihood ratio chi-square (degree of freedom = 1);

p (Exact):Exact test
